# Supplementary material for: Different Amounts of Water Supplementation Improved Cognitive Performance and Mood among Young Adults after 12 h Water Restriction in Baoding, China: A Randomized Controlled Trial (RCT)
Source: Int J Environ Res Public Health. 2020 Oct 24;17(21):7792. doi: 10.3390/ijerph17217792 (PMC7662706; doi:10.3390/ijerph17217792)
Supplement: Supplementary file 1 [file ijerph-17-07792-s001.pdf]

Supplementary Material

Table S1. The temperature and humidity of study days.

	Indoors		Outdoors	
	**Temperature (℃)**	**Humidity (%)**	**Temperature (℃)**	**Humidity (%)**
First study day	23.8	71	20.7	85
Second study day	21.8	70	17.9	93
